# How to construct an appropriate research design for the study of learner identity in blended learning?

**DOI:** 10.3389/fpsyg.2023.1126605

**Published:** 2023-01-19

**Authors:** Xiuping Lian, Xinmin Zheng

**Affiliations:** ^1^International College, Fujian Agriculture and Forestry University, Fuzhou, China; ^2^School of Education, Shanghai International Studies University, Shanghai, China

**Keywords:** learner identity, blended learning, online collaborative writing, research design, validity and reliability

## 1. Introduction

With English language becoming the norm for academic communication and knowledge exchange for scholars and students around the world, the focus of English as a foreign language (EFL) or English as a second language (ESL) has gradually shifted from English for general purposes (EGP) to English for academic purposes (EAP) (Li, 2020; Maswana and Yamada, 2021). EAP aims to cultivate learners' academic English language ability as well as to broaden their knowledge of disciplinary culture (Hyland and Hamp-Lyons, 2002; Jund, 2010; Douglas and Rosvold, 2018). Under this circumstance, learner identity in EAP education witnesses an increasing attention from researchers in the field (see Etherington, 2006; Charles and Pecorari, 2022). Some studies (Lave and Wenger, 1991; Sinha, 1999; Coll Salvador and Falsafi, 2010; Delahunty et al., 2014) reveal that the acquisition of new knowledge has an impact on individual's construction of learner identity in a given context. Compared with massive studies on learner identity constructed in traditional face-to-face classrooms (Trent, 2008; Wearmouth et al., 2011; Nasrollahi Shahri, 2018; Lan and Lan, 2022; Xu and Kim, 2022) or online learning (Ginns and Ellis, 2007; Mayrberger and Linke, 2014; Kwon et al., 2021), to date, research studies on learner identity constructed with blended learning method seem scarce and insufficient, attaching to EAP courses in particular.

Besides, it appears that few studies have examined learner identity from the perspective of dynamic process in collaborative writing. To be more specific, collaborative writing is frequently employed as a learner-centered approach to engage students in English learning because of the limited class hours set for the course (Chen et al., 2022; Lan and Lan, 2022). Abound with meaning of negotiation, the benefits of collaborative writing in facilitating interaction and development of basic language skills have been widely documented (Storch, 2005; Kim, 2008; Wigglesworth and Storch, 2009; Shehadeh, 2011; Dobao, 2014; Ahmad, 2020; Anggraini et al., 2020). In addition, learner identity construction in collaborative writing has also captured researchers' attention (see Arnold et al., 2012; King, 2015; Brown and Pehrson, 2019; Chen et al., 2021). Among the scarce studies on learner identity, researchers mainly focus on the linguistic elements in learners' written products rather than the dynamic process to examine learner identity in collaborative writing.

As teachers and researchers, we show our concern about the learner identity in blended EAP education, and we read with great interest the newly-published article entitled “*Exploring learner identity in the blended learning context: A case study of collaborative writing*” by Chen et al. (2022). For the sake of reader's convenience, we will refer it as “the article” hereafter. The article aimed to explore six Chinese university students' learner identities constructed in the online and offline collaborative writing sessions of an EAP course. It paid special attention to the construction of learner identity in relation to learners' engagement and interaction during the process of blended collaborative writing section. In our view, the article has bridged two above-mentioned gaps and contributed a conclusion with both interpretative and illuminating evidences, which demystified and specified the construction of learner identity and its influencing factors in a blended EAP course. We believe the article will provide researchers and practitioners with a new perspective of learner identity construction. Therefore, we would like to comment on the strengths and weaknesses of the article, especially focusing on the validity and reliability of research design, aiming to provide constructive suggestions for future relevant studies in the field. In order to better guide our analysis, we propose the following two research questions:

1. How did the article select participants in order to dig out more relevant data to address its research questions?
2. How did the article analyze the data to facilitate the in-depth exploration and understanding of the intricate phenomenon such as learner identity construction in relation to learners' engagement and interaction?

In our discussion process, we adopt content analysis method (Bell et al., 2022) to examine the selection of participants, data analysis and research findings of the article. Concerning the data analysis process in our article, first, we read the article recursively and independently. Then, we coded and categorized the description and discussion texts relating to the selection of participants, data analysis and research findings of the article. For the sake of trustworthiness, we compared each other's coding, discussed how to reach a consensus on different coding between us, and finally modified them until agreement and consistency were maintained. We also sought feedbacks from two expert researchers to solicit their suggestions for the purpose of triangulation.

Before we analyze the article in detail, we think it is necessary to review and explicate a key term in the article, namely, learner identity in order to help better understand and interpret the article. Some researchers (see Sampson, 1978; Burke, 1980; Scotton, 1980; Heller, 1984) interpreted learner identity as fixed personalities, learning styles, and motivations, but recent studies of learner identity adopt a dynamic approach, thus making a sharp contrast to those in the early stage. Coll Salvador and Falsafi (2010) made a distinction between learner identity and learner identity process (LIP), in which learner identity was a fixed image separating from learning situations, while LIP was a process that emphasized the experience and adaption of learner identity to a particular learning context. Based on a post structural perspective, Norton (1997, p. 410) defined identity as “how a person understands his or her relationship to the world, how that relationship is structured across time and space, and how the person understands possibilities for the future”. This definition was echoed by Flórez González (2018) who claimed identity was fluid, context-dependent, and context-producing, constructed in certain historical and cultural circumstances (see also Toohey, 2000; Pavlenko and Blackledge, 2004; Norton and Toohey, 2011; Nasrollahi Shahri, 2018; Lan and Lan, 2022).

In following sections, we will first provide a summary of the article, then discuss its advantages and limitations by addressing our two guiding questions, and finally summarize the implications of the article.

## 2. The study

Qualitative in nature, the article employed a case study approach to examine learner identity construction and its influencing factors in a blended learning context. Six non-English major male participants were selected from a comprehensive university based on an initial four-week observation due to their diverse engagement in learning activities.

In regard to the data collection, the article collected multiple sources of data derived from four collaborative writing sessions, including class observations, field notes, semi-structured interviews, history logs on the writing platform and the transcriptions of participants' offline group discussions for the purpose of triangulation.

With reference to the data analysis, the article examined the data that revealed the learner identity and their influencing factors inductively and deductively by classifying the data into online and offline categories. First, the data that expressed learner identity in offline collaborative learning sessions were analyzed by taking a discourse analysis method. Specifically, the article identified, coded and categorized participants' verbal characteristics in offline classroom discussions by drawing on Poupore (2016) analytical framework, followed with the statistics of frequencies of each participant's verbal characteristics. In this way, the article aimed to reveal learner identity separately. Second, the data that reported learner identities in online collaborative writing sessions were analyzed by adopting the framework of work load roles proposed by Arnold et al. (2012). Be specific, the frequencies of participants' writing revisions which acted as a main reflection of participant' online engagement were counted to investigate the learner identities in online sessions. Finally, two rounds of semi-structured interviews were transcribed, coded and categorized to examine the factors influencing learner identity construction in online and offline sessions.

The article revealed three major findings based on the data analysis. First, the article demonstrated that the construction of learner identity in blended learning depended largely on specific learning activities and learning contexts, with more positive identities in offline sessions and negative ones in online sessions. Such divergence may be caused by teacher's different involvement or pedagogical guidance in two learning sessions. In addition, the article also disclosed that both individual and contextual factors had an impact on learner identity, in which individual factors intersected to affect learner identity construction in both learning sessions, while the impact of contextual factors changed according to different learning sessions. Finally, the article illustrated that the learner identity construction displayed different patterns in online and offline sessions, with some participants demonstrating consistency and others revealing changes. This finding further proved that individual's learner identity was constantly confirmed and reconstructed through LIPs.

In the end, the article provided implications for course design, pedagogical practice, and materials development in blended learning context, appealing that a careful course design and teacher's active involvement or guidance are essential for maintaining learners' positive learner identities and improving learning outcomes.

## 3. Discussion

In this part, we would like to make comments on the selection of participants, data analysis and research findings, aiming to examine the validity and reliability of the article for the sake of facilitating further research in this field. In particular, by discussing the group size in the article, we attempt to arouse researchers' attention to the intention, representativeness as well as transparency of the selection of participants. In addition, comments on the data analysis intend to raise researchers' concern about the importance of analysis unit, data analysis triangulation as well as transparency of analysis process. Finally, by comparing the findings in the article with those in previous researches, we appeal that the first priority should be given to the selection of theoretical framework.

### 3.1. Selection of participants

The article picked a focal group with six participants demonstrating diverse engagement in learning activities based on an initial four-week observation. Obviously, the article selected the focal group intentionally, attempting to explore the relationship between learners' interaction and learner identity construction. Nonetheless, one thing that puzzles us is the size of group, since the article did not explicate the reason why the group consisted of six participants. In our opinion, it would be better to make an explanation about it, as many studies have revealed that the size of group has a tremendous impact on participants' involvement in group discussion (Mishra, 2016) and interaction in collaborative writing (Arnold et al., 2012; Dobao and Blum, 2013; Dobao, 2014). Therefore, we argue that future study should include groups of different sizes to make a comparison in order to have a deep and detailed understanding of the impact of learners' interaction on learner identity. As Tenny et al. (2022) indicate the more representative the sample is to the expected research population, the more likely the researcher will take various factors at play into consideration. We believe that the selection of participants should be purposeful (Sargeant, 2012) to saturate the data, and at the same time the details and processes involved in the selection should be elaborated for the purpose of transparency (Oun and Bach, 2014).

### 3.2. Data analysis

As far as we are concerned, the data in the article seemed to be clearly classified into online and offline categories and analyzed inductively and deductively with peer debriefing adopted to ensure the trustworthiness of the data analysis. However, after taking a close look at the analysis process of learner identity in online collaborative writing, we perceive some problems regarding the selection of analysis unit, reliability of analysis framework and transparency of the analysis process. First, drawing on the work load roles put forward by Arnold et al. (2012), the article utilized revision frequency as an analysis unit to identify learner identity without considering the type of revision, such as formal or meaning-based one. In reality, Arnold et al. (2012) discovered that learner identity transferred when different types of revision were taken into account, for learners made different efforts to revise different types of errors or problems based on their own perceived advantages and limitations. Therefore, we suggest that the type of revision should be a better choice for being an analysis unit, as data analysis aims to describe a phenomenon in detail in qualitative study (Flick, 2014).

In addition, we are skeptical about the reliability of the analysis framework adopted in the article as we discover that the criterion used to examine work load roles in the article is inconsistent with that in Arnold et al. (2012). Specifically, work load roles were judged by the workload of revision in Arnold et al.'s study, while it is determined by the frequency of revision in our commented article. As addressed by the authors themselves, the frequency of revision served as a proxy of students' online involvement (Chen et al., 2022, p. 6), which, in our opinion, is not equivalent to actual contributions of revision. Given the divergence mentioned above, we suggest that another analytic method be combined for researchers to eliminate bias and seek convergence among a variety of data to build up themes or categories (Golafshani, 2003). In other words, it is suggested that triangulation of data analysis methods be employed, if necessary, to make up for the weakness of a single technique and enhance the interpretation and reliability of research findings (Thurmond, 2001).

Finally, we find no clue as to how the learner identities are verified from the two rounds of semi-structured interviews for there is no description about the process of data analysis in this regard. In fact, the elaboration of analysis process is indispensable because data do not speak for themselves, it is the analyses and interpretations on the part of researchers that yield descriptive and causal inferences in qualitative study (Moravcsik, 2020).

Given the major influence of data analysis on research findings (Flick, 2014), we appeal that future research should give weight to the selection of analysis unit in that an appropriate unit is conducive to locate the data relevant to research questions (Mezmir, 2020). In addition, researchers should make sure that data analysis method selected is suitable for the study. If necessary, another analysis method can be combined for triangulation (Leech and Onwuegbuzie, 2007). Finally, the process of data analysis needs to be transparent and trustworthy (O'Kane et al., 2021).

### 3.3. Research findings

With regard to the research findings of the article, on the one hand, the factors that influenced learners' engagement and interaction in the online collaborative writing sessions are distinct from those in previous researches. When we examine the findings carefully, we find that only the individual (learners' English ability, character and perception) and contextual factors (assigned roles and teacher's involvement) are disclosed. However, previous studies have found that various factors may affect learners' engagement and interaction, such as the genre of writing (Reed et al., 1985), the type of task (Li and Zhu, 2017) and computer-mediated contexts (Wang, 2019). Moreover, different communication modes, for instance, using online conference or online editing software will elicit changes in learners' engagement and interaction in collaborative writing (Aubrey, 2022). Based on the description in the article and our investigation of writing platform, we figure out that the very limited interaction in online collaborative writing can partly attribute to the communication mode of platform, on which participants communicate mainly through text messages asynchronously. As mentioned by one participant in interview, it was difficult for group members to communicate on the internet platform, and it was challenging for new revisers to comprehend the previous one's intention (see Chen et al., 2022, p. 10). Therefore, future research could incorporate above-mentioned factors into online course design. This suggestion also echoes the finding that lack of a well-organized online learning session resulted in the transformation of a positive LIP into a negative one in blended learning (see Chen et al., 2022, p. 11).

On the other hand, no macro-level factors related to learner identity construction were discovered. In fact, many macro-level factors, such as race and culture (Kubota and Lin, 2009), societal power relations (Norton, 2013), educational policy (Hajar, 2017) have a significant influence on learner identity. We assume that the inconsistency of influencing factors can be ascribed to different focuses and organizations guided by theoretical frameworks adopted. It is obvious that the theoretical framework employed in the article mainly focused on learner identity in particular learning context. To the best of our knowledge, as a social being, learner identity is not only contextual constructed but also historically, culturally and politically situated (Pavlenko and Blackledge, 2004). Therefore, an alternative, for instance, the framework proposed by Norton (1997) could be adopted to examine both micro and macro influencing factors. In fact, the selection of theoretical framework is vital because it is the base for the construction of knowledge (Osanloo and Grant, 2016) and provides an anchor for analysis and interpretation of data as well as research findings (Merriam and Tisdell, 2016). As a result, we argue that researchers should give their first priority to the selection of theoretical framework for it not only determines the focus, organization, exposure and hiding of meaning in the study, but also relates the study to previous scholarship and concept (Collins and Stockton, 2018).

## 4. Conclusion

In summary, the article is a thought-provoking, well-explored, and illuminative piece. Firstly, it expounds the interplay of learner identity construction and learners' social interaction. In addition, the process of collaborative learning activities rather than the static writing product is examined. Finally, the patterns of learner identity construction across different learning sessions are revealed. All in all, the article provides readers with new insights into the complexity of learner identify and variety of influencing factors. We believe that, after reading the article, course administrators, teachers and students can have a better understanding of learner identity and factors that hinder or facilitate positive learner identity construction in blended learning context, which prompts them to make corresponding adjustments in their course design, teaching or learning respectively. Hence, we would like to recommend the article without any hesitation to more readers, particularly those who are keen on learner identity in blended learning.

## Author contributions

XL and XZ selected the commented article together. XL drafted the opinion. XZ provided insights and valuable suggestions during her writing and helped revise the text. All authors contributed to the article and approved the submitted version.
